# Parental Concerns and Active Participation in Home-Based Vojta Therapy for Children with Global Developmental Delay: A Qualitative Study Using Interviews and Photo-Elicitation

**DOI:** 10.3390/healthcare14010104

**Published:** 2026-01-01

**Authors:** Ana San-Martín-Gómez, Carmen Jiménez-Antona, María Salcedo-Perez-Juana, Livia Gomes Viana-Meireles, Domingo Palacios-Ceña

**Affiliations:** 1 Research Group of Humanities and Qualitative Research in Health Science (Hum&QRinHS), Department of Physical Therapy, Occupational Therapy, Physical Medicine and Rehabilitation, Universidad Rey Juan Carlos, 28922 Madrid, Spain; ana.sanmartin@urjc.es (A.S.-M.-G.); maria.perezjuana@urjc.es (M.S.-P.-J.); domingo.palacios@urjc.es (D.P.-C.); 2 Institute of Physical Education and Sports, Universidade Federal do Ceará, Ceará 60455-760, Brazil; liviagviana@ufc.br

**Keywords:** Home-Based Program, parents, Vojta, developmental delay, qualitative research, photo-elicitation

## Abstract

**Highlights:**

**What are the main findings?**

- Parents reported that home-based Vojta therapy facilitated parent–child affiliative bonding.
- Parents in this study indicated that adherence to HBP-delivered VT was perceived as beneficial and sustainable despite their initial concerns.

**What are the implications of the main findings?**

- Given appropriate information and resources, more parents of GDD children would choose to administer home-based Vojta therapy.

**Abstract:**

**Introduction:** Parents of children presenting global developmental delay (GDD) need to be involved in their therapy to intensify treatment. Vojta therapy (VT) is an intensive physiotherapeutic treatment that can be administered at home. Whilst parental experience of Home-Based Program (HBP) for preterm or cerebral palsy is well documented, there is a lack of understanding about parents of GDD children on HBP with VT. **Objectives:** The aim of this work was to describe parents’ perspectives concerning their participation in, concerns with, and perception of the results of an HBP with VT. **Methods:** A qualitative case design based on an interpretative approach was presented. A purposeful sampling was used. Data was collected in two stages: firstly, semi-structured interviews, and secondly, photo-elicitation. An inductive thematic analysis was used. **Results:** Seventeen parents were included. Three themes emerged from parents’ perspectives. Firstly, parents’ active participation in VT, which includes their desire to become an active agent to contribute to their child’s improvement, their implication of compromise, learning process, time required, effort, and factors that influence their adherence and continuity. Secondly, parents’ perception of the results achieved: motor improvement and better resting, feeding, and breathing; and time and commitment required to achieve them. Thirdly, parents’ initial concerns about suitability, daily implementation, therapy functioning, or evidence, as well as concerns about emotional bonds. **Conclusions:** Parents universally perceive that their commitment and efforts were rewarded. They recognized that the emotional bond with their child was strengthened by the therapy. The results regarding the beneficial effects perceived by the parents should be treated with caution, as no instruments for assessing the effect or efficacy were used in this study.

## 1. Introduction

Global Developmental Delay (GDD) is a neurodevelopmental disorder characterized by the delayed acquisition of two or more developmental milestones [1]. Early intervention is key to reducing the negative impact of developmental delay [2,3].

Increased hours of daily therapy are reportedly associated with greater gains in motor function [4,5]. The effectiveness of intensive physiotherapy is closely linked to principles of neuroplasticity: repetition, intensity, specificity, and training [6]. To optimize frequency and repetition of intervention, parental participation is needed [7]. Consequently, incorporating family involvement in intervention is recommended for favorable neurodevelopmental outcomes in the early therapy setting [8].

Increased daily exercise is possible on Home-Based Programs (HBPs) [5]. HBPs are therapeutic activities designed for children to perform with parental assistance in the home environment [9]. In order to effectively administer HBPs, parents undergo training with the support and coaching of a qualified therapist [10,11].

Given the fundamental importance of frequency and consistency of application, VT is an ideal candidate for home-based delivery. VT is a neuromodulative treatment based on reflex locomotion principles that aims to promote typical innate patterns [12]. It involves the application of pressure to specific stimulation points to activate innate automatic movement patterns [13]. This pressure stimulation induces a specific somatosensory response that triggers innate, stereotypical motor patterns, thereby modulating central motor control mechanisms [14] and improving motor function in individuals with neurological or developmental disorders, especially infants and children with motor delays [15,16]. VT is designed to harness and promote neuroplasticity by providing structured sensory input that facilitates the reorganization of motor pathways [17]. Nevertheless, the effectiveness of VT in the pediatric population is subject to criticism. In this way, Sanchéz-González et al. [17], in their systematic review and meta-analysis on VT effectiveness, stated that, in adult patients, effects were observed on cortical activity, balance, and muscle activity in VT groups, compared to the control groups, whereas no significant differences in the pediatric population were found when gross motor function, respiratory rate, and height were assessed. In that same study, they suggest its potential usefulness for the treatment of respiratory, neurological, and orthopedic conditions [17].

Parents must be trained in the delivery of VT [16]. This training is imparted in physiotherapy sessions [12] in which an experienced physiotherapist illustrates the VT techniques, instructing the parents on their execution and on the kinesiological responses expected [18]. Parents are taught key points such as maintaining symmetry during exercises, ensuring proper stabilization of support points, and positioning the head correctly. Parents practice under direct supervision to ensure they can accurately apply VT at home [12]. In addition, parents receive detailed instructions for therapy sessions to be repeated at home, four times each day [16,18,19,20]. Comprehensive guidance, both written and in person, is provided [12], ensuring that VT follows the key HBP features of learning and supported skill development.

Given the high level of commitment required of parents in delivering VT in the home, their perceptions and experiences must be heard. It is well documented how parents of preterm or cerebral palsy children deal with HBP [21,22,23,24]. However, there is a lack of understanding about parents of GDD children undertaking HBP with VT.

This study aims to describe parents’ perspectives and experiences concerning participation in, and adherence to, an HBP with VT, as well as their perceptions of results and concerns with a HBP with VT.

## 2. Materials and Methods

### 2.1. Design

A case qualitative study was conducted, based on an interpretive framework [25]. A case qualitative study is a useful design for describing the experiences of professionals, patients, and families in the developmental medicine field and child neurology [26,27], and to understand the level of acceptance or rejection of treatments.

Five researchers participated in this study (4 women), including one researcher nurse (DPC), three physical therapists, all of whom are child neurology experts (CJA, ASMG, MSP), and a psychologist (LGVM). All researchers work at the University. Background context of the application of VT in Spain is described in Table S1 [12,13,16,17,19,20].

### 2.2. Participants, Sampling Strategies, and Recruitment Process

**Figure 1:**
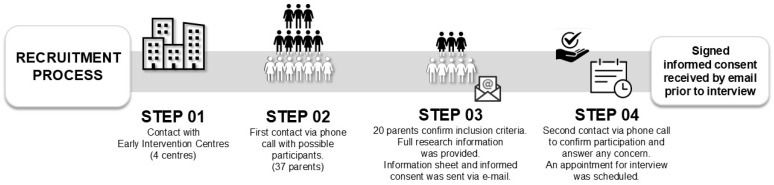
Participant recruitment procedure.

A purposeful sampling was used [28]. Parents of GDD children being treated with VT were recruited from four Early Intervention Centers (EICs). Potential participants were contacted through the EIC’s physiotherapists (see Figure 1).

Inclusion criteria consisted of (a) parents of children diagnosed with GDD (without an identified metabolic, genetic, or neurological cause) by neurologists’ diagnostical test (code 315.8 (F88) DSM-V) [1]; (b) being receiving VT from a trained professional with a recognized certification from the Spanish Vojta Association (www.vojta.es); and (c) being undertaking VT to their GDD child at home more than 3 times a week [29] at least for 2 months before being recruited for the study. Exclusion criteria were parents of GDD children who perform VT ≤ 3 times a week. Vojta therapy’s characteristics and recommended treatment regimen [16,19,20] are described in Table S1, in the context of Vojta therapy.

There are different proposals for estimating sample size in qualitative research (pragmatic considerations, saturation, power of information, etc.) [30], as there is no previous formula for calculating it [28]. Previous studies using empirical tests determined the number of interviews necessary to achieve saturation [31,32]. A saturation proposal by Hennink and Kaiser [32] based on an empirical test was applied. It describes that data saturation is achieved between 9 and 17 interviews (with a mean of 12–13 interviews) in qualitative studies where the objectives of the study are narrowly defined, and the populations under study are homogeneous (participants shared and possessed direct information about the phenomenon under study). With this proposal, a greater capacity to identify codes, categories, and topics is achieved. In addition, the current proposal also helps researchers to know a reference number to stop collecting data and/or recruiting participants. A universe of potential participants was initially contacted (n = 37), but due to the unavailability of many cases, the authors of the present study established the sample size based on pragmatic considerations [30]. All available cases were included to obtain a greater richness of the data. Previous qualitative studies on the perspective of parents of children with disabilities or neurodevelopmental disorders using photo-elicitation (PE) used sample sizes between 10 and 15 parents [33,34]. Finally, seventeen parents were included. The main reason for not participating was the limited time available (n = 9), non-adherence to treatment (n = 4), and lack of interest in the research (n = 7).

### 2.3. Data Collection

**Figure 2:**
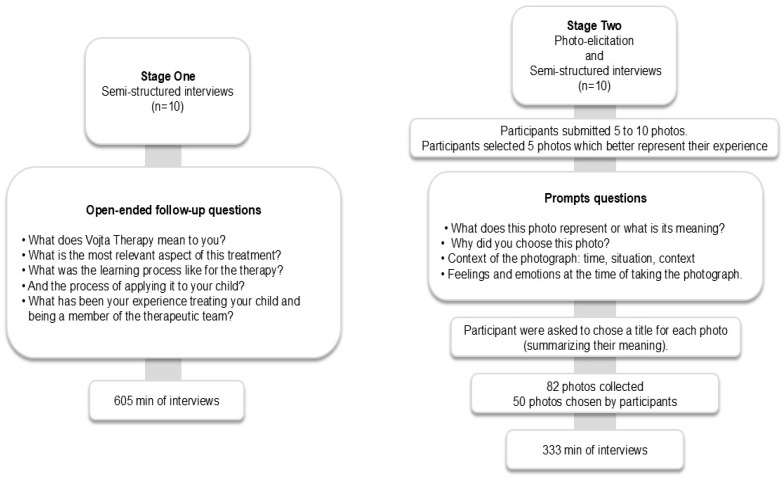
Data collection procedure.

Data was collected in two distinct stages. Stage One comprised individual semi-structured interviews using a question guide. In this stage, ten parents participated. Thereafter, the researchers noted the key words identified in the participants’ responses and used their answers to ask them to clarify the content [28] (see Figure 2. Data collection procedure).

Stage Two involved photo-supported interviews using the PE technique. Participants submitted photographs of their choice, including them in the qualitative interview process [35,36]. With PE, each participant interprets their photographs and their meanings for the researcher [37,38,39]. This helps the researcher to understand the full breadth of the participant’s experiences (including emotions, feelings, and ideas) rather than imposing the researcher’s framework [40]. This stage involved ten parents, including three carried over from Stage One and seven newly enrolled participants. A total of three participants took part in two interviews, one interview conducted in Stage One and the other in Stage Two. Stage Two participants received guidelines for taking photographs using their own mobile phones. The photographs were sent to researcher ASMG, and a date was set for a semi-structured interview with researchers ASMG, CJA, and LGVM. In the interview, participants selected the five photos that they felt best represented the application of VT at home. The researcher used four prompt questions to guide the PE interview. Additionally, participants were asked to create a title for each photo to summarize its meaning to them. A total of 82 photographs were submitted, of which 50 photographs were chosen by participants as those best representing VT in the home. See Figure 2.

In both data collection stages, the interviews were conducted via a private video chat room using the Microsoft Teams platform (https://www.microsoft.com/es-es/microsoft-teams/log-in (accessed on 14 October 2023)). Each participant received a private and personalized email with an invitation. All interviews were conducted by two researchers (ASMG, CJA, and LGVM). Additionally, the researcher’s field notes were used as a secondary source of information.

### 2.4. Data Analysis

All interviews were transcribed and submitted to inductive thematic analysis [41]. In Stage One, inductive analysis was conducted, generating codes which were reviewed by the researchers. Researchers then developed categories from these codes, and following discussion, consensus was reached on final themes.

**Figure 3:**
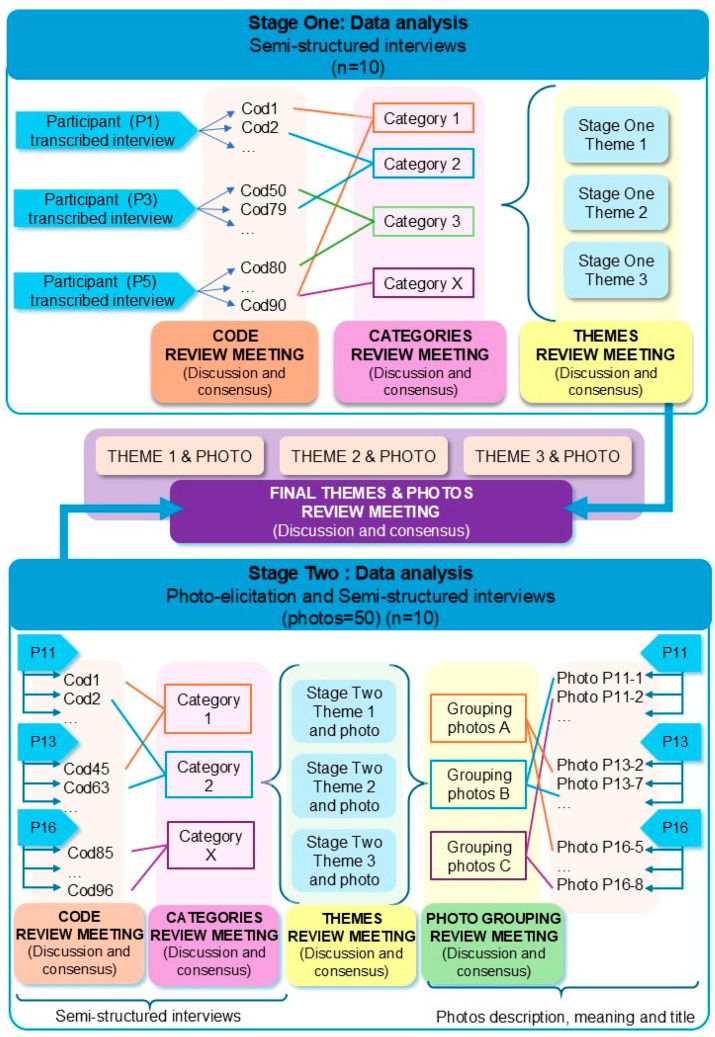
Data analysis procedure.

A similar codification process was applied to the Stage Two interviews. Also, to confirm, triangulate, deepen, and enrich Stage Two findings, an analysis of narratives related to the photographs was made, with photos being grouped based on their title, description, and meaning. Themes were derived from Stage Two, based on a textual content analysis of the participants’ narratives regarding the meaning and representation of the images used. Furthermore, no discrepancies were identified between images and narratives. Figure 3 depicts this data analysis procedure.

In both stages (Figure 3), codification, categories creation, and themes determination were established through periodical research team meetings, in which results were discussed, and in case of disagreement, it was resolved by consensus. Throughout the analysis process, matrices were developed using Microsoft Excel to better understand the relation between emerging themes and participants’ images [41]. Eventually, with all the qualitative data from both stages, a new meeting was held to reach a consensus, which identified three final themes. Every theme was linked to specific quotations and the corresponding photographs. No qualitative software or Artificial Intelligence was used.

### 2.5. Rigor

The procedures used to control trustworthiness are described in Table 1 [27,42]. Also, the Standards for Reporting Qualitative Research (SRQR) [43] and the Consolidated Criteria for Reporting Qualitative Research (COREQ) were followed [44].

### 2.6. Ethics

This study adhered to the principles of the Declaration of Helsinki. This study received ethical approval from Ethic Committee of Universidad Rey Juan Carlos (code: 3006202326423). All participants provided written informed consent (a) to be included in the study; (b) for the researchers to publish or otherwise disseminate narratives obtained in the study; (c) for the researchers to use photographs obtained in the study (Stage Two participants only); (d) for the researchers to use photographs for articles, subject to prior elimination of any identifying data and anonymization of the material via an alphanumeric code. Written informed consent was collected before enrolment in the study and data collection. Recorded video-audio interviews and participants’ photographs were anonymized.

## 3. Results

Seventeen parents of GDD children were recruited (six male). Their mean age was 38.47 years (SD 4.58). Three following themes emerged: (a) Parents’ active participation in VT, with three categories (desire to became an active agent; implications of undertaking VT at home; adherence to and continuity of the HBP); (b) Participants’ perception of results; and (c) Parents’ initial concerns with two categories (initial concerns, implementation and evidence; emotional bond). Table 2 shows participants’ demographic information and children’s age.

Narratives that justify and confirm the traceability of the results [42] are shown in Table 3, Table 4 and Table 5.

### 3.1. Parents’ Active Participation in VT

This theme encompasses the parents’ perspectives regarding being an active participant and the implications of undertaking VT at home.

#### 3.1.1. Desire to Become an Active Agent

VT requires implementation several times a day, every day of the week, which represents a significant commitment. Several participants felt the HBP with VT furnished them a welcome opportunity to contribute to their child’s improvement, expressing thankfulness for this opportunity and taking comfort from feeling useful. Participants also perceived that just one session of VT per week is not enough; therefore, in their opinion, parents’ daily participation at home is needed.

Participant EP1 took a photo of her hands and her child to illustrate her commitment and the hope/joy that her hands were important tools for her child’s development. From the participants’ point of view, they described their intervention within the therapy as beneficial due to their perception of children’s rapid improvement. Feelings of shared success and pride were present.

#### 3.1.2. Implication of Undertaking VT at Home

While appreciating the feeling of usefulness and their perceived progress, parents also recognized the level of hard work involved. Participants universally acknowledged VT at home as a “job,” a responsibility, a chore, or an obligation that contributes to their child’s improvement. The job was defined as hard and extensive. In fact, a participant described it as a “slavish task”.

Participants discussed the significant effort involved in the learning process, the ongoing concentration required, and also referred to the associated stress, with psychological and time demands being the most significant. Regarding time commitment, they described it as a daily investment made in their child for which they expected to see positive returns. To illustrate this, participant EP5 chose a screenshot of his diary as one of his 5 representative photos. He explained his need to schedule VT into his workday, emphasizing the significant daily time commitment that VT represents—a widespread opinion among participants, some of whom submitted photos of watches. Additionally, some parents felt frustrated if they were unable to fulfill the full VT schedule on a given day.

#### 3.1.3. Adherence and Continuity

Despite the significant efforts involved, parents universally regarded VT as it is a worthwhile, referring to their participation as “rewarding” and “a help to carrying on.” Some participants emphasized they would do it again without hesitation, despite the significant effort involved. Three participants described it as a guide to understanding their child’s development, a step to follow, and a way of moving forward.

Participant EP11 submitted a photo of a lawn dart, representing how “VT helped her to move forward, achieving new goals, and signing to her child’s recovery.”

All participants indicated their consistency was linked to their perception of VT’s results. Some parents were motivated to undertake VT at home by observing their achievements during their weekly session with the Vojta physiotherapist. Several participants commented on the direct relationship between time commitment and results perception.

**Table 3:** Narratives of theme 1: Parents’ active participation in VT.

Category: Desire to become an active agent
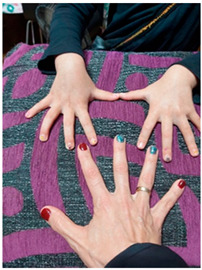 *Title: “Here are our hands to work as a family”* EP1-Photo 6 *“This image conveys that to me, like a balance, like a strength, right? (…) of being there and of offering, here are our hands to work for whatever you need.” (EP1)* *“I think it is a positive thing because, in the end, you get involved. Only once a week with the physiotherapist…., with this, being part of it, well, you force yourself to participate throughout the week as well, right? It also makes him improve a lot faster because instead of doing it once a week, maybe he is doing it 3 or 4 times a week, and you feel part of the improvement.” (P6)* *“(my son) He could be better…then, if there is anything I can do something, so he is even better, I do it. So, she (the therapist) taught me how to do it right, it took me time to learn it. When I learned how to do it right, I felt confident, and my baby got the greatest improvement; he moved much better.” (P2)* *“I felt responsible for my son’s improvement.” (P5)* *“As you see, it’s working for him, you feel responsible when you don’t do it., right? “It’s a little bit of how poorly I organized myself today that I didn’t manage to do it.” I feel a little bit of frustration.” (EP4)*
Category: Implication of performing VT at home
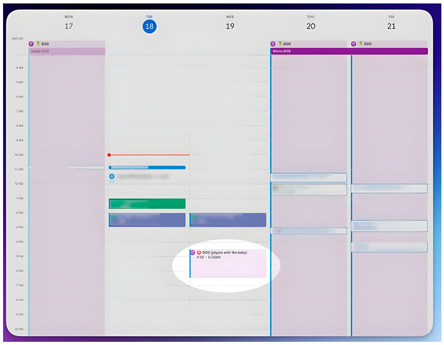 *Title: “Conciliation” EP5-Photo 1* *“I chose this photo because it represents how much I need to block out time from my work, umm… to invest in Vojta therapy and in (child name), in my son.” (EP5)* *“It is much more extensive, it enslaves you, so to speak, it requires much more time, but well, Vojta is for me a method and a way of stimulating my baby that gives very good results and very quickly.” (P2)* *“Well, for me it involves me, a lot of patience, (…) “I have to do this, I have to do it right.” First, be calm, include in your daily life, at least… it is almost 3 h or 45 min multiplicated by 3 to dedicate… THAT TIME TO THAT GIRL, that time to that girl, because it is important… for her.” (P14)* *“To me, it means work, perseverance, dedication, effort, discipline, perseverance, it’s ultimately the working tool with which I can apply Vojta therapy to (child name).” (EP15)* *“For me, the job is to organize it and then do it, but I also see that (child name), the more you work with him, the better he is.” (P1)*
Category: Adherence and continuity
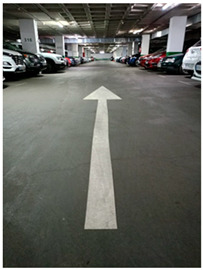 *Title: “Knowledge and information” EP11-Photo 2* *“Look for some way of moving forward, of representing… the way of expressing what you have in your head (…) Well, that’s what it is for me, that “I am capable of moving forward”, with Vojta…Yes, really, eh::: going little by little, achieving, achieving new things each time, reaching goals, little by little and, advancing more (…), taking steps towards recovery.” (EP11)* *“It worked for us. That…it’s a pain because you know? She cries a lot. But… that… that’s it, It’s worth it. And that’s it” (P7).* *“If we hadn’t seen improvement in a few weeks, we would have stopped, but when we started to see it after a while, at first it was a very small improvement, (…) and then we saw a very clear evolution.” (P8)* *“It means continuing the benefit you get from Monday sessions and being able to continue until the following Monday.” (P1)* *“I would never define myself as my son’s physical therapist! I’m more like Berta’s (physiotherapist) hands when Berta’s not around, right? We’re more of a remote physical therapist. She does the thinking, and we do the hands a little.” (P4)*

### 3.2. Participants’ Perception of Results

This theme gathers parents’ perspectives regarding the results of VT. The number of hours of therapy is positively perceived by parents. However, individual cases displayed variation in the benefit perceived–acquisition time profiles. Some participants narrated improvements from the first VT session, which encouraged them to implement it at home. However, other participants reported no immediate effects but observed slow progression over the course of the HBP, defining VT as “a long-distance race.” Parents described that VT requires consistency to perceive results, but the effects perception is varied, cannot be predicted, and improvements cannot be guaranteed.

Parents described some improvements in their children, such as leg and arm movements, gripping, strength, with head control and gaze tracking standing out. Daily changes were described as subtle gestures, better tummy position, more active play and socializing, trying new things, and performing effectively previously difficult movements. Additionally, changes in resting, feeding, and breathing were perceived as an unexpected bonus to continue.

To illustrate this, participant EP13 photographed her child in its cradle at night. She explained how, following their first VT session, her child could finally sleep.

Some parents reported that the improvements they observed did not continue after discontinuing VT, and several participants even speculated that no progress would have been made without the therapy. Participants commonly recognized VT as a helpful method that they perceived as developmental changes in their children, despite initial concerns.

**Table 4:** Narratives of theme 2: Results perception.

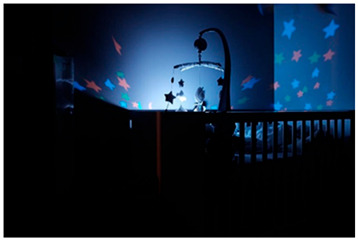 *Title: “Peace of mind that everything is working” EP13-Photo 5* *“So, when I finally took her to Vojta Physio for the first session. We arrived at night, everything was normal, everything was the same, and suddenly the girl…uh… falls asleep and doesn’t wake up until the next morning at 8:00 a.m., or on top of that, 12 h of sleep, which we’ve never seen before. “What?” My husband expressed it like this, “Just for this session… it was worth it,”… “if we go back to normal tomorrow, he says, I don’t care, but it was worth it.” (EP13)* *“Know that until you have applied the therapy for a while, you will not see any results. It’s a long-distance race.” (P5)* *“It’s also true that he used to eat better and sleep better after Vojta’s therapy, as he gets tired a lot, so that was a plus for us, (…) Well, the truth is that was also a plus, wasn’t it? He continues sleeping, continues sleeping better. And eating even better.” (P3)* *“Well, for me it was one of the first milestones we achieved, and it made me feel like we were making important progress (…) for me the fact that I was starting to open my hands and that I was starting to manipulate objects quite well, well, that was the sensation… That, happiness too.” (EP3)* *“Every time we do the… therapy afterwards, we try to put him over on his tummy to see if that time spent like that was actually worth it, right? Then the child just lifts his chest more.” (EP16)* *“But if you invest in your child doing the therapy, then you’ll see results that perhaps you wouldn’t see otherwise. I’m not sure that…that it wouldn’t have worked without Vojta, but I do think that when we don’t do Vojta, she’s weaker, I mean, I do see a difference. In fact, when she’s sick, which has happened to us twice now, and we can’t do Vojta, you can tell she’s weaker.” (P9)* *“We, well, now I’m telling you that it’s starting, we’re starting to see changes that we understand are due to development, but the child wasn’t holding his head up until a month ago. And… And yes, we believe it’s because of Vojta, well. Very clearly, right? Because we’ve been seeing improvement in the exercises. On the sides we’ve…. It’s something that can be seen.” (P4)* *“Ah, well the work that we do in therapy when we go to the sessions or the… and the homework that they give us to take home and what you work on with… with the child, well you see that, Uh, he’s achieving things that… that were difficult for him, it’s a little bit more, well he’s moving his neck better, he’s making more movements with… with the part, it was the upper part, especially the one that he had the most problems with and… And you see him, well little by little he’s achieving certain… certain milestones and certain moments that… that of course maybe without therapy, well we wouldn’t have achieved them.” (EP11)*

### 3.3. Parents’ Initial Concerns

This theme collects parents’ perspectives regarding their initial concerns about VT implementation, the evidence supporting it, and its potential effect on emotional bonds with their GDD children.

#### 3.3.1. Initial Concerns, Implementation, and Evidence

Skepticism about the effectiveness of VT was an initial concern, with some participants having doubts about whether it was the right therapy for their child. The lack of published research and medical consensus increased their concerns, as did the absence of VT from the “menu” of potential treatment options at major hospitals and the scarcity of physiotherapists expert in VT.

Concerns about effectiveness persisted in the early days of treatment, although confidence increased as results were observed. Parents found that VT was not easy for beginners. During the implementation of VT at home, participant insecurity showed through in many cases. Clear instructions from Vojta physiotherapists, together with videos and drawings, were fundamental to their learning and confidence. Participants related how the Vojta therapists were resolving concerns during sessions, and their support was recognized and highly regarded. All parents felt more confident as long as they improved their performance on VT.

To illustrate this, participant EP5 photographed his child with the Vojta therapist on the mat at the start of a VT session. He explained how the therapist starts every session by playing with the child and observing his progress and verbalizing each facet of this progress to the parents. He highlighted the bond the therapist had created with his child and how this built confidence in VT.

#### 3.3.2. Emotional Bond Concerns

All participants expressed concerns about potential damage to the emotional bond. Some participants related an initial fear of harming their child while undertaking VT. Participant EP17 photographed her child’s foot. She explained how she had to push on her child’s foot during VT. Given the repetitive nature of VT, this meant that she had to push on her child’s foot three times a day, every day, leading her to worry about potential bruising.

In contrast with this negative feeling, several parents expressed gratitude. They considered undertaking VT as an opportunity to create stronger bonds with their child. VT involved three sessions a day of physical contact, and a sense of working together for progress. These participants were able to recognize their child’s effort and strength and felt proud to be a part of the improvement perceived.

**Table 5:** Narratives of theme 3: Parents’ initial concerns.

Category: Initial concerns, implementation, and evidence
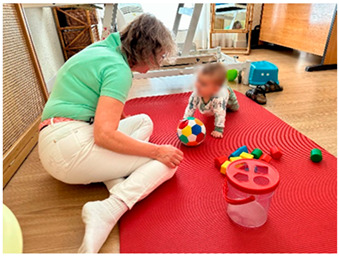 *Title: “Seeing you” EP5-Photo 5* *“Vojta therapy, too, it must be said, is, in my case, tough. My baby cries, cries a lot, and… and that increases my anxiety, increases my anguish, increases my doubts about, like, “Uh? Whether it’s the right thing to do or not?” Or what other therapy might work for him?” (P2)* *“At that moment, you think: “Am I doing it right? Am I doing it right? Are you breaking it?” In that state, you are like this, like nervous, but…, but no, she (the therapist) did reassure us, “Look, you are doing this right, put it a little more like this, a little more like that”, it is like in that moment you are more concentrated and::::and:::: listening to her and her advice” (P8)* *“Well, on the one hand, amazed, right? You ask yourself, “How, how did Mr. Vojta come up with this?” Of course, because he has a learning curve, a learning curve that I don’t have behind me. Right? “But how did he manage to press these keys and see what produced this?” But I’m surprised that the hospitals don’t… Don’t move it (VT) anymore. Because it’s not like they don’t have Vojta people, I mean, no. It’s not like they didn’t put Vojta in ours because they think he’s very good, it’s just that no, there aren’t people who do Vojta in general. So we were surprised.” (P4)* *“Well, hey, it takes time, first to learn it, then to gain a little confidence.” (P14)* *“(At home) we have doubts, so, hey, is he crying because I’m taking away her movements? Or is he crying because I’m not doing something right? And on top of that, could I end up hurting her? Right? Because no, because I don’t know how to do it right.” (EP16)* *“This is (name child) in the clinic, before starting Vojta exercises with Berta (physiotherapist). He’s on all fours, crawling, playing some games for (name child), but we also, well (…) it’s good for us to appreciate (name child) ‘s crawling. Berta tells us certain aspects that (name child) can improve, (…) she tells us, “look at this and see how (name child) is crawling now,” but it also helps (Child name) play a little, to warm up a little before the session, and also to appreciate each other, right? So also, to improve that bond with Berta, since they both get along well, (…) I always really like this little bit of time before starting the exercises. (EP5)*
Category: Emotional bond concerns
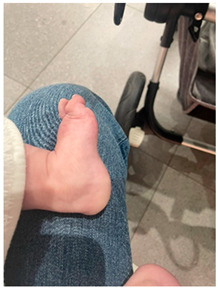 *Title: “Tiny Feet” EP17-Photo 7* *“Well, initially, a lot of doubts. I’m a high school teacher, so, uh, I’m not much of a psychology teacher, but I did have some basic knowledge, and I remember that the first few days, I became obsessed with the subject of attachments and bonds. “Oh, my goodness, how is this going to affect the attachments that I myself am causing her at the moment of annoyance?” (P4)* *“I don’t know how that could affect him. Whether she’ll be left with some trauma or not, or whether she’ll forget.” (P9)* *“A concern I might have is that she’ll be traumatized by the image of “When I was little, my father was on top of me, you know? Squeezing me with his thumbs.” (P7)* *“In therapy, you have to squeeze (the foot), learn to squeeze it well, the points, (…) for us it is a learning process, because we are not physiotherapists, (…) to do it well, so that it does not cause more damage or more pain than perhaps, well, unconsciously we can do it. Look, it is supposed that the normal thing is that it does not hurt; he must feel uncomfortable to achieve the result, but it does not have to hurt.” (EP17)* *“Myself as the mother, whoever does the Vojta therapy made me feel very special, very connected to my baby.” (P2)* *“I really appreciate it a lot, having been able to know…This therapy because…because you get so involved.” (P1)* *“Well, that’s it, in the end, I am his father, right? And I want the best for him…, I have seen the difficulties he had when he was born, so contributing to this whole process of improvement*, etc., *It is important to me, because that way I feel more…part of the whole process and…and as I say, it’s not like I sit and watch a specialist do X exercises and nothing more, but I get involved…, I am part of the process. it also makes me create more of a bond with… with (Child name), I spend more time with him…,* etc. *So that’s why it’s important to me, yes.” (P5)* *“But I think it’s because of the relationship that’s created, right? With your son, because in the end, you’re helping him. He notices that you’re helping him. And right? Well, a moment you have alone with him, then, I don’t know exactly if only a professional would do it, you’d have the same ties, but having to participate, and right? To be a co-participant in all this therapy, well, obviously, you have a greater bond than with another person, I mean, more than if you just take him to the doctor. (P6)* *“And so, well, I’m always surprised by the amount of sweat he produces, and you can see the effort it takes to do the exercises, right?” (EP5)*

## 4. Discussion

Family involvement in HBP is fundamental. Parental implementation of flexibility exercises, neuromotor development training exercises, body mechanics, and postural stabilization exercises at home [45,46,47,48,49] has been shown to produce benefits perceived by parents, as children with GDD achieve new milestones, improved posture, and/or greater social participation and adherence to treatment [45,50,51]. Previous research also shows that parents who participate in HBP delivery of therapy benefit from a feeling of usefulness and empowerment [46,47,52].

Despite parents’ commitment to undertake home treatment [45,47,51,52], some negative emotions can emerge due to the weight of responsibility related to treatment application and prescription (intensity, time, frequency, etc.) [46,53].

Previous research conducted on parents of children with disabilities and developmental delay who undertook HBP [48,49,54] highlighted five factors that hindered parental adherence to HBP: (a) the perception of complexity of the exercises which requires great skill from parents, (b) the significant amount of time required to administer the therapy, (c) the high frequency of therapy sessions (once a day, from three to five sessions per week), (d) the sense of personal burden, and (e) the perception of discomfort in the child. On the one hand, our results aligned with these previously identified factors, with our participants also describing VT as difficult to learn, requiring a significant daily and weekly investment of time, and initially having concerns about their child’s discomfort. Although in previous studies [48,49,54] performing more than three sessions per week in BPH could be a factor that decreases adherence, this phenomenon did not appear in our participants. On the other hand, some parents reported having perceived results with this therapy in their children, which allowed them to continue with their commitment to their child’s treatment.

In HBP, parents must apply and repeat the regimen of exercises prescribed by the professional physiotherapist, which may mean applying the treatment several times a day, several days a week [45,46,47,48,49,52,54]. The participants in this study perceived that consistency is required to obtain results. Parental time investment has been previously studied [46,47,48], highlighting the significant effort parents must make to organize their work life around the treatments they administer. Several participants submitted photographs of diaries or clocks representing the time pressures arising from HBP.

Previous research has indicated that parents taking an active role in HBP-delivered treatment of children with disabilities or developmental delay is crucial [45,46,47,48,49,50,51,52,53,54]. Our results reveal, as a new insight, that the parents described having an active role in their children’s treatment as a positive experience. This collaborative role is not gratuitous; it requires a significant investment of time, concentration, and learning complex content. It also entails parents submitting themselves to a great deal of responsibility when actively providing treatment to their children in the solitude of their own homes. These results reveal new lines of research into the reasons and factors for adherence to HBP with VT in this group of parents.

Enhanced improvement in their child’s development is every parent’s desire when undertaking an HBP [45,46,47,52]. Previous studies reported how the improvements perceived by parents are a determining factor for continuing and adhering to therapy [45,46,48,49,50,52,54]. Consequently, it is necessary to highlight the role of professional physiotherapists in teaching parents to recognize these improvements and the advances made by the refs. [46,47,48,50,52]. Diverse studies show that parents gain confidence both in administering treatment and in recognizing the effects and changes in their children. Thus, time is a key factor in empowering parents [45,46,47,48,49,50,51,52,54]

Previous research into VT-based treatment of children with developmental delay [16,19,55,56,57] showed improvements: better posture [16,19,55,56,57], better head control and gaze tracking [19], increased strength [55], increased activity, increased capacity for movement, improved sociability [50,57], upgrades in feeding as rhythmicity and regularity of sucking improves [58,59], and enhanced breathing [60,61,62,63]. However, many of these studies have small sample sizes, are case reports, or lack randomization, limiting the high-quality evidence on VT. In addition, Sánchez-González et al. [17], in their systematic review and meta-analysis of the existing evidence on VT in adults and children, highlighted the scarcity of robust randomized controlled trials for VT in pediatric neurodevelopmental disorders. Nevertheless, these authors [17] conclude that although the current evidence supporting VT is of limited quality, there are indications suggesting its potential usefulness for the treatment of respiratory, neurological, and orthopedic conditions.

The aim of this study was to describe parents’ perspectives about their active participation in an HBP with VT in their children, not to quantitatively evaluate therapy’s effectiveness. On the other hand, qualitative research helps to understand the use of therapies with low evidence, due to its capacity to explore meanings, sociocultural contexts, and dynamics among professionals, patients, and families [64,65]. Thus, qualitative research allows us to study how practices and discourses are constructed around different therapies and to describe the beliefs, values, and subjective experiences of patients and professionals who choose these therapies [65,66,67].

Our results, based on the parents’ perspective, showed that applying therapy involved an emotional, time, and dedication effort. This raises an ethical debate about the application of therapies with limited evidence and their potential unintended effects on families, such as increased stress, decreased quality of life, and financial burden [68]. Families of children with disabilities are a vulnerable population, and therapists should support families in their therapeutic decision-making process, informing them about the evidence for different interventions [68]. Paleg et al. [68] point out that intensive therapy models must be carefully analyzed to assess the opportunity cost of their application for the child. Applying these interventions in high doses can represent a sacrifice for the child (missing school attendance, social activities with friends), limiting their experiences and their educational, emotional, and social well-being.

The concerns of parents starting HBP about the suitability of the treatment, whether they are applying the treatment correctly, or whether they might cause harm or discomfort to their child, have been previously described [45,46,48,52]. Previous studies have also shown how the presence and support of a professional during HBP is essential to achieving parental self-efficacy [45,48,52]. Furthermore, several authors [45,46,48,49,54] have reported that the parents’ fear of causing harm/discomfort to their children during HBP caused some parents to abandon the therapy. In this study, the guidance provided by Vojta therapists, through developmental teaching and explanations of how the therapy works, helped address these uncertainties and foster parental adherence and self-efficacy.

Another new insight that our results revealed was that carrying out the therapy together (father/mother–child), frequently (three times/day), and seeing the effort and dedication of their children, their struggle, and their progress, had improved the parent–child bond between participants in our study. Since no previous studies were found by the authors that have described or analyzed the development of this bond during HBP, it is impossible to determine whether it is related to the specific practice of Vojta therapy at home or whether it is a feeling shared by all parents who support their children at HBP.

### Limitations

In qualitative health research, justifying sample size remains challenging, where determining a priori sample size is often not feasible [30,69,70]. On account of the two-stage data collection and analysis process, which employed different data collection instruments (interviews and photo-elicitation) and different types of qualitative data (text and images), the iterative process for data saturation could not be achieved. Therefore, the authors used empirical testing as the sample size criterion to determine a reference number of interviews needed to saturate the data and a pragmatic approach, including all available participants, considering the difficulty in accessing participants [69]. Secondly, VT is highly sensitive to the technique of the professional applying it, so variability in its application between centers or therapists could influence parents’ perceptions when learning and applying the treatment to their children. To avoid this, the authors only included parents whose children were receiving VT from a professional accredited by the Spanish Vojta Association. Thirdly, the use of the GDD diagnosis by the DSM-5 is not a specific diagnostic category and can be caused by many etiologies. On the one hand, the lack of a specific etiology may influence parents’ perspectives, as perceived improvements can vary depending on their children’s underlying diagnosis. On the other hand, the percentage of children diagnosed with GDD without a determined cause reaches 62% [71]. In addition, it is recommended that children with GDD be referred to early intervention, even without knowing the etiology, to avoid delaying early intervention [72,73,74]. Therefore, it would not be possible to compare perceived improvements among participants. Although the aim of this study was not to compare parents’ experiences but to describe them all, the results related to children’s improvement (theme 2) due to VT should be considered with caution, in particular two participants who were receiving other therapies (speech therapy and stimulation), and in these cases, the description these two parents made cannot be certain that this progress perception is solely due to this treatment.

Finally, participants were recruited exclusively from centers where HBP with VT was administered. This could bias the results toward a positive view of the treatment. Notwithstanding, in the qualitative methodology, participant selection is aimed at obtaining relevant information to answer the proposed study objective (non-probability sampling) [27,28]. The researcher’s expectations, directing questions to obtain specific answers, and when participants seek to please the researchers, could arise as a positive bias in qualitative methodology. As a result, information may be interpreted, recorded, or presented in a more favorable way [75]. The following strategies are recommended in the study ref. [76] to avoid or minimize this bias: (a) Triangulation (multiple sources and methods); (b) Reflexivity (the researcher acknowledges their influence); (c) Use of open and neutral non-directive questions; and (d) Member checking. All of these strategies have been included in the present study.

It should be pointed out that this study was not conducted to measure VT efficacy and effects in HBP; rather, it aimed to describe the parents’ perspective on the therapy and its treatment modality. Qualitative research helps to understand people’s use of therapies through their ability to explore the meanings, beliefs, values, subjective experiences, and dynamics among patients, their families, and professionals in the applications of these therapies [64,65].

## 5. Conclusions

With adequate training and support from a professional therapist, parents can overcome initial concerns and conclude that their significant commitment to HBP-delivered VT is rewarding. The findings of this study reveal the key issues that should be considered when prescribing HBP-delivery of VT. In the relevant process of shared decision-making, clinicians should provide balanced information about the time commitment, lack of high-quality efficacy evidence, and potential psychosocial impacts—both positive (empowerment, bonding) and negative (stress, burden)—they may experience during treatment. One novel aspect of our results is the emotional bonds between parents and children who perform HBP with VT in their children with GDD. However, the results on the effect of VT should be handled with caution because the results show the (subjective) experiences of parents of children with GDD who receive VT in HBP, without using any assessment or instrument to evaluate the effect or efficacy of the therapy.

In addition, developing educational programs aimed at addressing the implications for parents undertaking HBP with VT is essential. Future lines of qualitative research could be developed to further explore the emotional dimension of therapeutic experiences implemented in the family context with other therapies.

## Figures and Tables

**Table 1:** Trustworthiness criteria.

Criteria	Techniques Performed
Credibility	Researcher triangulation: Each interview was analyzed by two researchers. Subsequently, team meetings were held in which the analyses were compared, and themes were identified.
	Triangulation of data collection methods: Semi-structured interviews were conducted, participant-driven photographs were taken, and the researchers made field notes.
	Member checking: This consisted of asking participants to confirm the data obtained during data collection. All participants were offered the opportunity to review the audio and/or video recordings to confirm their experience. None of the participants made any additional comments.
Transferability	In-depth descriptions of the study were made, detailing the characteristics of the investigators, participants, sampling strategies, and data collection and analysis procedures.
Dependability	Audit by an external researcher: An external researcher evaluated the study’s research protocol, focusing on aspects related to the methods applied and the study design.
Confirmability	Researcher and data collection triangulation. Researcher reflexivity was encouraged through reflective reporting.

**Table 2:** Participants’ demographic information and children’s age profile.

Participants	17 participants (6 fathers, 11 mothers)
Age, years (parents)	Mean: 38.47 years (SD 4.58)
Work situation	Work active: n = 11 Leave of absence: n = 4 Parental leave: n = 2
Number of children	Only child: n = 8 More than one child: n = 9
Educational status	College education: n = 17
Time within Vojta therapy (in months)	Between 2 and 3 months: n = 9 Between 3 months and 1 year: n = 8.
GDD child’s age.	Less than 6 months old: n = 5 More than 6 months and less than a year old: n = 9 Older than a year: n = 3

## Data Availability

The data presented in this study are available on request from the corresponding author due to ethics and legal restrictions.

## Supplementary Materials

The following supporting information can be downloaded at https://www.mdpi.com/article/10.3390/healthcare14010104/s1, Table S1: Context of Vojta therapy application in Spain.

## Abbreviations

The following abbreviations are used in this manuscript:

EIC	Early Intervention Centers
GDD	Global developmental delay
HBP	Home-based program
VT	Vojta therapy
PE	Photo-elicitation
