# Supplementary material for: Parental Concerns and Active Participation in Home-Based Vojta Therapy for Children with Global Developmental Delay: A Qualitative Study Using Interviews and Photo-Elicitation
Source: Healthcare (Basel). 2026 Jan 1;14(1):104. doi: 10.3390/healthcare14010104 (PMC12786262; doi:10.3390/healthcare14010104)
Supplement: Supplementary file 1 [file healthcare-14-00104-s001.zip › healthcare-4054682-supplementary.pdf]

Supplementary material S1.
Table S1. Context of Vojta therapy application in Spain.

Vojta therapy stimulation is performed in a defined starting position (Refle Rolling in the supine and side lying position, and reflex creeping from the prone position) [17] with specific pressure stimulation points on the body [*13*]. This stimulation is implemented on left and right side, twice each side [12]. According to VT recommendations, session duration typically ranges from 5 to 20 min per session, four times a day, every weekday [16, 19, 20]. VT with parents' participation is therefore an intensive physiotherapy linked to neuroplasticity principles.

To apply Vojta Therapy (VT), it is necessary to have complete training, since therapist must be able to identify child's weaknesses and strengths, know what immediate achievements are needed to continue with their development, plan the therapeutic intervention knowing how to choose the most recommended exercises, have the teaching and training capacity to be able to teach and involve parents in the execution of these exercises at home, as well as correct, modify and encourage parents to achieve correct adherence to treatment.

Vojta Therapy training in Spain is regulated by the Spanish Vojta Association (www.vojta.es) and offers international certification endorsed by the International Vojta Association (www.vojta.com). This training requires 320 hours of instruction. (https://vojta.es/xii-curso-de-terapia-vojta-en-alteraciones-motoras-infantiles-un-exito/)

Theoretical training involves rehabilitation physicians, psychologists, neuropsychologists, and physiotherapists. It covers the following topics (https://www.vojta.com/en/further-training/courses) :

- Human ontogenesis
- Postural reactions in their ideal and abnormal gestalt
- Dynamics of the primitive reflexes
- Biological basis to innate locomotion
- Reflex locomotion: reflex creeping, reflex rolling
- Facilitation
- Functional anatomy, particularly regarding locomotion
- Muscle function differentiation
- Movement analogies between reflex locomotion and motor ontogeny
- Kinesiological analysis of normal and abnormal motor development
- Assessment of the quality of movement in spontaneous motor activity
- Reflex locomotion as a therapeutic principle
- From pre-speech to verbal communication
- Analysis and evaluation of the automatic postural control of the spine as the basis of goal-directed movement
- The development of infantile cerebral palsy (ICP)
- Epilepsy in children with ICP
- The indications for surgery in ICP
- Signals and behavior in human relationships
- Opportunities and limitations in treatment using the Vojta Principle
- Training your perception, precision of self-awareness
- Stimulation zones, guiding points of resistance, treatment techniques
- Interaction with the patient under the principle of attentiveness

- Individual responsibility of the therapist and patient
- The wide range of treatment applications, daily therapy
- Aspects of the provision of assistive devices
- Parent instruction: Training in family intervention, communication, emotional management, positive leadership, listening to parents, and collaborative planning of objectives and the therapeutic plan.

This training methodology includes:

- Theory is practice-based and analytical
- Practical exercises
- Functional analysis
- Demonstrations of treatment
- Treatment of patients by the course participants under supervision of Vojta Instructors
- Self-experience

The practical training encompasses the assessment of over 200 patients and their families on Spanish Public Hospitals. Students participate in guided practice with instructors, focusing on communication with the family and child, assessment, goal setting, treatment planning, treatment implementation, and teaching parents how to monitor progress. This includes addressing parents' questions, providing parents with homework exercises, facilitating practice and correction, and offering appropriate feedback.

Upon successful completion of the course, students receive a certificate in "Developmental Kinesiology Course, according to Vojta, applied to infants, children, and adolescents with motor impairments," which accredits the holder as a qualified and internationally certified Vojta therapist. (www.vojta.es) and Continuing Education Commission for Health Professions of the Community of Madrid, National Health System. (https://vojta.es/xii-curso-de-terapia-vojta-en-alteraciones-motoras-infantiles-un-exito/)

Correct application of VT requires performing the prescribed exercises three to four times a day, every day of the week. To achieve this, families visit the early intervention centre once a week, while parents apply the therapy at home the rest of the time, i.e., several times a day. This requires a process of teaching and learning VT from therapists to parents. The learning process takes place during the sessions, setting realistic goals, teaching defined starting positions, trigger zones defined in location, quality, intensity, and duration, the activation of innate genetic motor patterns as desired responses, and the correction of unwanted ones [*12*]. This learning is continuous, and it is advisable to ask parents about any questions or difficulties during each session in order to modify and refine the intervention at home.

Parents are essential agents for the success of VT, as they must be present during their child's treatment, plan realistic goals, learn the exercises, apply them at home, and be able to make modifications guided by the professional.
